# Analysis of Operant Self-administration Behaviors with Supervised Machine Learning: Protocol for Video Acquisition and Pose Estimation Analysis Using DeepLabCut and Simple Behavioral Analysis

**DOI:** 10.1523/ENEURO.0031-24.2024

**Published:** 2025-02-03

**Authors:** Leo F. Pereira Sanabria, Luciano S. Voutour, Victoria J. Kaufman, Christopher A. Reeves, Aneesh S. Bal, Fidel Maureira, Amy A. Arguello

**Affiliations:** ^1^Department of Psychology, Michigan State University, East Lansing, Michigan 48823; ^2^Department of Earth and Environmental Sciences, Michigan State University, East Lansing, Michigan 48823

**Keywords:** DeepLabCut, operant behavior, pose estimation, predictive classifiers, Raspberry Pi, self-administration, Simple Behavioral Analysis (SimBA), video recording

## Abstract

The use of supervised machine learning to approximate poses in video recordings allows for rapid and efficient analysis of complex behavioral profiles. Currently, there are limited protocols for automated analysis of operant self-administration behavior. We provide a methodology to (1) obtain videos of training sessions via Raspberry Pi microcomputers or GoPro cameras, (2) obtain pose estimation data using the supervised machine learning software packages DeepLabCut (DLC) and Simple Behavioral Analysis (SimBA) with a local high-performance computer cluster, (3) compare standard Med-PC lever response versus quadrant time data generated from pose estimation regions of interest, and (4) generate predictive behavioral classifiers. Overall, we demonstrate proof of concept to use pose estimation outputs from DLC to both generate quadrant time results and obtain behavioral classifiers from SimBA during operant training phases.

## Significance Statement

Substance use disorders are comprised of complex behaviors that promote chronic relapse. Rodent operant self-administration is commonly used as a preclinical tool to examine drug-taking, drug-seeking, and drug-craving behavior. We provide protocols to acquire videos of self-administration behavior and obtain pose estimation outputs and unique behavioral classifiers using the supervised learning softwares DeepLabCut and Simple Behavioral Analysis.

## Introduction

Substance use disorders (SUDs) are comprised of a set of behaviors that impair quality of life (Saunders, 2017). Patients diagnosed with SUDs can remain abstinent for extended periods of time, but exposure to drug-associated stimuli often triggers intense craving and a return to drug use (Gawin and Kleber, 1986; Ehrman et al., 1992). Rodent operant self-administration is used as a preclinical tool to examine drug-taking, drug-seeking, and drug-craving behavior. Rats are implanted with jugular catheters to allow for intravenous (IV) delivery of a substance and undergo distinct phases of behavioral training. During cocaine self-administration (Coc-SA), responses on an active lever result in cocaine infusion (paired with a tone and light, or in a unique context), whereas responses on an inactive lever result in no consequences. During extinction (Ext) training, responses on both levers result in no consequences (Fuchs et al., 2008; Perry et al., 2014; Li et al., 2016). During a reinstatement, or Relapse Test, responses on the active lever result in the presentation of the drug-paired cue (or occurs in the previously cocaine-paired context) without drug reward—with increases in responses considered an index of drug-seeking behavior (Fuchs et al., 2008; Perry et al., 2014; Venniro et al., 2021; Arguello et al., 2024).

Several open-source tools allow for fine-tuned analysis of behavior from video recordings. Supervised machine learning models such as DeepLabCut (DLC), Social LEAP Estimates Animal Poses, or DeepPoseKit allow for analysis of behavioral videos using pose estimation (Mathis et al., 2018; Graving et al., 2019; Pereira et al., 2022; Luxem et al., 2023). DLC extracts frames from video recordings, the user labels key body parts, and these labeled frames are used to train a convolutional neural network (CNN; Mathis et al., 2018). This supervised machine learning approach has been used to obtain pose estimation from behaviors such as trial-based nose poke and operant self-administration training, among others (Clemensson et al., 2020; Goodwin et al., 2022; Centanni and Smith, 2023; Iglesias et al., 2023; Isik and Unal, 2023). Pose estimation data can then be used as the input for predictive classifiers which utilize random forest algorithms such as Simple Behavioral Analysis (SimBA; Goodwin et al., 2024). Given that lever responses are primarily used to examine drug-taking and drug-seeking behaviors, it is critical to incorporate additional behavioral information to more comprehensively interpret taking and relapse behaviors. We provide protocols to acquire behavioral videos with Raspberry Pi (RasPi) or GoPro cameras, obtain pose estimation outputs with DLC and a high-performance computer cluster (HPCC; https://icer.msu.edu/web-portal-hpcc-resources), and analyze pose estimation data with SimBA to generate predictive classifiers of behavior during specific phases of contextual operant training: cocaine self-administration, extinction, and relapse tests.

## Materials and Methods

### Animals

The study utilized male Sprague Dawley rats (Envigo). Videos were collected for *n* = 16 rats; however, a total of *n* = 7 was used for final analysis due to issues with initial video acquisition, such as incorrect positioning of field view, truncated videos, or missing frames, which prevented their use in subsequent training steps. Rats were housed in pairs in a controlled environment with regulated humidity and temperature, under reverse lighting conditions, habituated, and handled for a period of 6–8 d before undergoing jugular catheterization surgery. Following a postrecovery period, rats were individually housed and switched to restricted feeding conditions as previously described (Cho et al., 2020; Charpentier et al., 2023; Olekanma et al., 2023). All protocols were approved by the Institutional Animal Care and Use Committee at Michigan State University (MSU) and adhered to the National Research Council's Guide for the Care and Use of Laboratory Rats.

### Surgery and behavior

Rats underwent surgery, with catheters implanted into the right jugular vein. Rats were fully anesthetized via intraperitoneal (IP) injection of ketamine and xylazine (80–100 mg/kg and 5–10 mg/kg, respectively), with appropriate pre- and post-analgesia care, and monitoring assessed (Charpentier et al., 2023; Olekanma et al., 2023). In the current study, video recordings were obtained during the Coc-SA phase of behavioral training for one cohort of rats (*n* = 3). Videos obtained from a second cohort of rats (*n* = 4) included Coc-SA, Ext, and Relapse Test behavioral phases. Rats underwent training in operant chambers (29.5 cm × 24 cm × 28 cm) configured with distinct odor, tactile, auditory, and visual stimuli. Context A contained a white house light (0.4 fc brightness), intermittent pure tone (80 dB, 1 kHz; 2 s on, 2 s off), pine-scented air freshener, and wire mesh flooring (26 cm × 27 cm). Context B contained an intermittent white stimulus light over the inactive lever (1.2 fc brightness; 2 s on, 2 s off), continuous pure tone (75 dB, 2.5 kHz), vanilla-scented air freshener, and a slanted black acrylic panel bisecting the bar flooring (19 cm × 27 cm). In addition to lighting within the operant box, diffuse red lighting was added within the sound-attenuated chamber to facilitate improved quality of video recordings.

**Figure 1.**
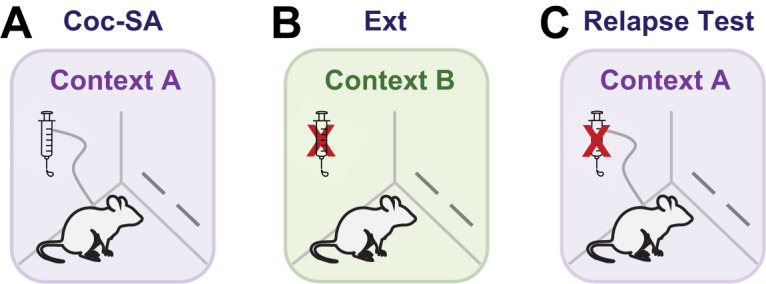
Schematics for each behavioral phase. All experiments included ***A*** cocaine self-administration training (Coc-SA, 2 h sessions, minimum of 10 sessions) in a distinct context, followed by ***B*** extinction training (Ext, 2 h sessions, minimum of 8 sessions) in a second context. ***C***, Rats were returned to the previous cocaine-paired (Coc-pair) context during Relapse Tests (2 h session).

Rats that started Coc-SA training in Context A received Ext training in Context B and vice versa (Fuchs et al., 2007; Cho et al., 2020; Olekanma et al., 2023). Coc-SA training occurred during daily 2 h sessions for a minimum of 10 sessions (criteria, at least 10 infusions). Active lever responses resulted in an IV infusion of 0.05 ml of cocaine hydrochloride (HCl, 0.5 mg/kg per infusion) on a fixed-ratio 1 schedule of reinforcement, with no change in background stimuli upon drug infusion. Inactive lever responses resulted in no consequences, as previously described (Cho et al., 2020; Olekanma et al., 2023). Cocaine HCl (NIDA Drug Supply System) was dissolved in sterile saline. Drug delivery was controlled by an infusion pump (Med Associates; Model PHM-107), and each infusion lasted for 2 s with a 20 s time-out period. Ext training consisted of daily 2 h sessions for a minimum of eight sessions (criteria, <25 responses on the last 2 sessions). During Ext training, responses on the active and inactive lever had no programmed consequences. During the Relapse Test, rats were returned to the original cocaine-paired (Coc-pair) context for a 2 h session during which both active and inactive lever responses resulted in no programmed consequences; therefore, active lever responses served as an index of drug-seeking behavior (Fig. 1). We routinely utilize the contextual self-administration model to assess the impact of diffuse environmental stimuli (rather than explicitly paired cues) on drug-seeking behaviors (Fuchs et al., 2008; Cho et al., 2020).

#### Video acquisition protocol

**Figure 2.**
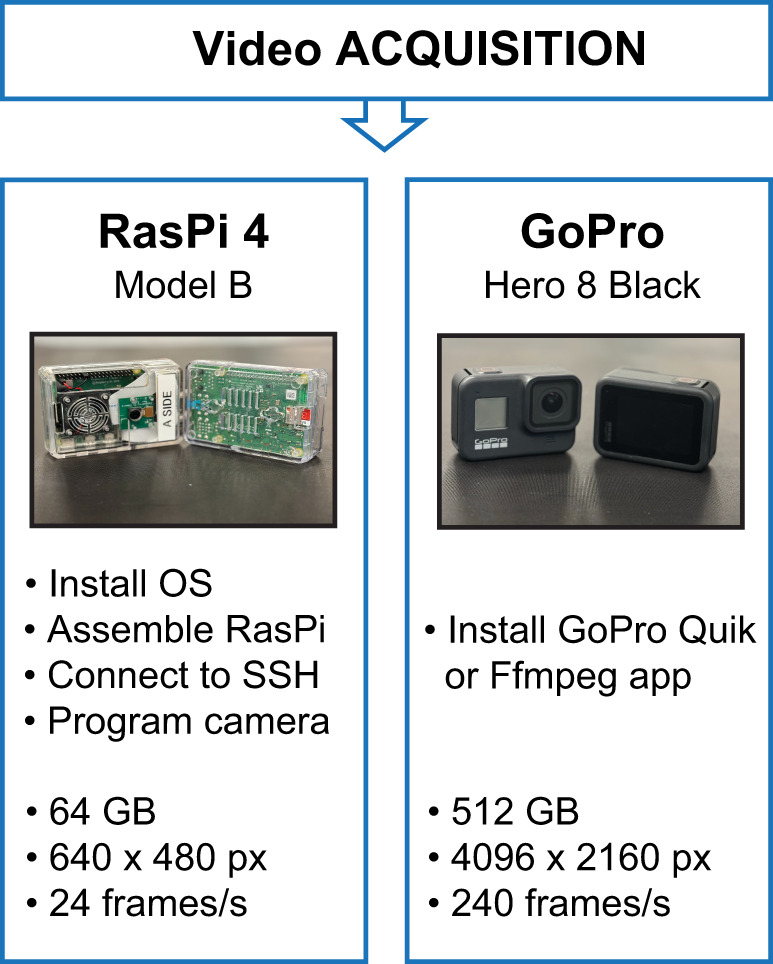
Video acquisition with Raspberry Pi (RasPi) or GoPro cameras. RasPi camera involves steps to install and assemble RasPi 4 microcomputer and camera for video recording, and install Termius and SSH to control the camera. The RasPi 4 (Model B) has a storage limit of 64 GB with a maximum video resolution of 640 × 480 px and 24 frames/s. The GoPro installation requires less steps, but a GoPro premium subscription or use of Ffmpeg open-source software is needed to process videos. The GoPro (Hero 8 black) has a storage limit of 512 GB with a maximum resolution of 4,096 × 2,160 px and 240 frames/s.

The following protocol outlines the steps to acquire videos using RasPi 4 microcomputers or GoPro cameras (Fig. 2). For RasPi, additional steps including setup with local Wi-Fi, Termius secure shell (SSH) installation, and programming RasPi Camera are outlined below. A list of materials needed to set up video recordings is provided in Table 1. Bold and italicized text represents code that must be input. Pointy brackets inside bold and italicized code ***<…>*** designate fields which must be replaced with text that will vary across instances of the protocol. The brackets should not be included as part of the fields (e.g., ***ssh <username>@<IP address>*** could be typed as ***ssh labuser@123.456.7.890***). Unless not in bold and italicized, include periods shown in lines of code.

**Table 1.** Materials required to setup Raspberry Pi (RasPi) camera and GoPro for video recordings

Product	Vendor	Catalog ID	Notes
RasPi Zero 2 W	Vilros	RPZW2	
SanDisk Extreme microSD	Western Digital	SDSQXA1-128G-AN6MA	1 h of video ≈ 1 GB. Storage of at least 32 GB is recommended
Official micro USB power supply for RasPi 1/1+/2/3/3+ and Zero-White	Vilros	SC0623	Any power supply with a micro USB connector, 5 V and 2 A
Official RasPi Camera Module V2	Vilros	RP_CAM_V2	RasPi Camera V3 allows for enhanced definition and wide-angle option, but does not fit the Zero Case
Official RasPi Zero or Zero W Case	Vilros	SC0049	Includes a short, Zero-compatible camera cable
Wi-Fi Router	Various	n/a	Ethernet cable and cable modem, if needed
GoPro Hero 8 Black	B&H Photo Store	n/a	

Suggested vendors are noted.

**Table 2.** List of useful commands to control, program, and transfer files from Raspberry Pi (RasPi)

Task	Terminal commands	Notes
Control RasPi	***ssh<Pi username>@<Pi IP address>***	Control of RasPi with Termius
	***cd ∼***	Change working directory to home directory
	***git clone*** https://github.com/reevesc7/RasPi-filming-code.git	Create a copy of RasPi-filming-code Git repository
	***sh RasPi-filming-code/setup.sh***	Run script to create recording commands
Transfer RasPi videos	***ls/media/<Pi username>***	Output ID of flash drive connected to RasPi
	***ls recordings***	Returns a list of all video files on the RasPi
	***cp –vr recordings/media/<Pi username>/<drive ID>/.***	Copies files from RasPi to flash drive
	***ls/media/<Pi username>/<drive ID>/recordings***	Displays a list of video files on flash drive
	rm recordings/*	Deletes all original video files on the RasPi
Shut down RasPi	***sudo shutdown –h now***	Powers off the RasPi

##### Assemble and power the RasPi

This section provides steps to assemble the RasPi computer.

1. Insert the microSD card into the RasPi. Ensure that the card's metal contacts are facing toward the metal contacts of the port.
2. Locate the RasPi's camera port on the opposite edge of the board from the microSD card slot. Carefully pull out the black lock until it loosens.
3. Insert the end of the camera's ribbon cable into the port. Face the metal contacts on the cable toward the white side of the port with metal contacts embedded in it.
4. Firmly seat the cable in the port while being careful not to bend it and press the lock down to secure it.
5. Place the RasPi in the bottom portion of the case. Ensure the ports line up with the proper cutouts.
6. Fit the camera into the camera-compatible lid of the case, align the camera into the circular hole of the case lid, and attach and close the case.
7. Plug the power cable into the RasPi, switch it on, and a red light should appear.

Note: To prevent data loss, do not depower the RasPi if it is on. Shut down the RasPi via the terminal using ***sudo shutdown -h now*** (see below, Shut down RasPi).

##### Access the RasPi via remote terminal

This section provides steps to control the RasPi remotely via a Wi-Fi–enabled PC.

1. Connect to the router with a PC.
2. Obtain the RasPi IP address, open internet browser, and enter the local IP address of the router into the address bar. A network map of all devices connected to the router should appear in the web dashboard; note the RasPi's IP address.
3. On the PC, open a terminal window (Command Prompt in Windows, Terminal in macOS) and type ***ssh<Pi username>@<Pi IP address>***. In the password prompt, type the password for the RasPi's user account. Once verified, the remote terminal can be used to control the RasPi.

##### Program the RasPi

This section outlines steps to download the script to control the camera. After programming the RasPi, the recording command needed to run the RasPi will automatically run upon SSH connection.

1. Access the RasPi via remote terminal.
2. Type ***cd ∼***.
3. Type ***git clone*** ***https://github.com/reevesc7/RasPi-filming-code.git***. To ensure successful download, typing ***ls*** should output the directory named “RasPi-filming-code.”
4. Run the setup script by typing ***sh RasPi-filming-code/setup.sh***.
5. A prompt to create a recording command will appear. Type this command into the terminal to initiate recording.
6. The setup script will ask whether the current command should be run upon SSH connection. In the terminal, type ***yes***. For creating additional recording commands and editing existing recording commands, see the Create, edit, and cancel a recording command subsection.
  Note: The duration of recording is the length, in seconds, that the RasPi will record. We recommend using a length that is longer than the duration of a behavioral session to ensure the entire session is captured.

##### Set up on-click SSH connection

This is to initiate SSH connections via the raw ***ssh*** command for more than one behavioral session. Termius (v5.20) is a free application which stores known host IP addresses, usernames, and passwords and can be useful for this step. Installing Termius requires an Internet connection, but the application can be used offline to connect to hosts if the host is either connected to the same Wi-Fi network or via Ethernet cable.

1. Create a Termius account on the PC used to access the RasPi.
2. Download the Termius installer by selecting “Get the apps” on the web app left menu.
3. Once installed, log in and select “Hosts” in the left menu and then “NEW HOST” at the top of the page, which will open a menu on the right.
  - Fill in the RasPi's IP address in the “Address” field.
  - Type the preferred name for the server in the “Label” field.
  - In the SSH section, type the username and password used to log in to the RasPi in the corresponding “Username” and “Password” fields.
4. Under “NEW HOST,” a new bubble with the newly created label should appear; double-clicking will open an SSH terminal interface connected to the RasPi.

##### Initiate RasPi video recording

1. On the PC used to access the RasPi, start Termius. On the “Hosts” tab, double-click on the host RasPi, and a terminal should open.
2. After typing the subject ID (with no spaces in between), press “Enter.” Now recordings can be initiated before behavioral sessions.

Note: If a prompt for the subject ID does not appear after the terminal opens, the recording command needs to be run.

##### Transfer Videos from the RasPi

Both wired and wireless transfer methods can be used to transfer video files from the RasPi, depending on the number and length of videos collected.

Flash drive transfer:

1. Insert a flash drive into one of the USB-A ports on the RasPi.
2. In the SSH terminal, type ***ls/media/<Pi username>*** to output the ID of the drive.
  Note: To get a list of all video files on the RasPi, type ***ls recordings***.
3. Type ***cp -vr recordings/media/<Pi username>/<drive ID>/***. to copy the files.
4. The original files on the RasPi can be deleted with rm recordings/*. Once done, the flash drive can be unplugged.

Wi-Fi network transfer:

1. While on the same Wi-Fi network as the RasPi, open a terminal, and enter scp<Pi username>@<Pi IP address>:∼/recordings/* <Path to local storage location>. The path for local storage can be inserted into the terminal by opening the folder in a file browser and dragging the folder into the terminal.
2. Enter the RasPi user's password when prompted for files to copy.

##### Shut down the RasPi

1. Connect to the RasPi via SSH.
2. Enter ***sudo shutdown -h now*** into the terminal to cancel the SSH connection. A green blinking light on the RasPi board will appear.
3. Once the green light stops blinking, the RasPi can be safely turned off.

##### Create, edit, and cancel a recording command

To cancel, cut short, or exit a recording command, “Ctrl+C” can be used. To create additional recording commands, type ***sh RasPi-filming-code/addcommand.sh***. If the user enters a command name that exists, permission to override the old command will be needed. To set existing commands to be run on SSH connection, type ***sh RasPi-filming-code/addcommand.sh***, enter the existing command's name at the first prompt, enter ***no*** to skip overwriting, and enter ***yes*** to set the named command as the one which automatically runs upon SSH connection. For useful command abbreviations, see Table 2.

##### Initiate GoPro recording and transfer videos

1. Install Adobe Premiere Rush app or Ffmpeg software for video editing.
2. Check settings on the camera (resolution, 1,920 × 1,080 px; frames/s, 24).
3. Ensure the camera is plugged into a power source and turned on before the start of behavioral session.
4. Transfer videos from GoPro SD card onto cloud or computer storage.

Note for RasPi and GoPro video acquisition: It is critical to perform test recordings to ensure post stitching and video processing do not impact the quality and/or length of videos. A mismatch in video length and frame number will cause errors and difficulty in obtaining regions of interest (ROIs) or predictive classifier data. We recommend that videos be transferred daily and organized in folders by behavioral phase with RatID and dates clearly noted. If the camera is removed from the power source, check that dates and times are accurate.

#### Protocol to acquire pose estimation data with DLC (key steps)

**Figure 3.**
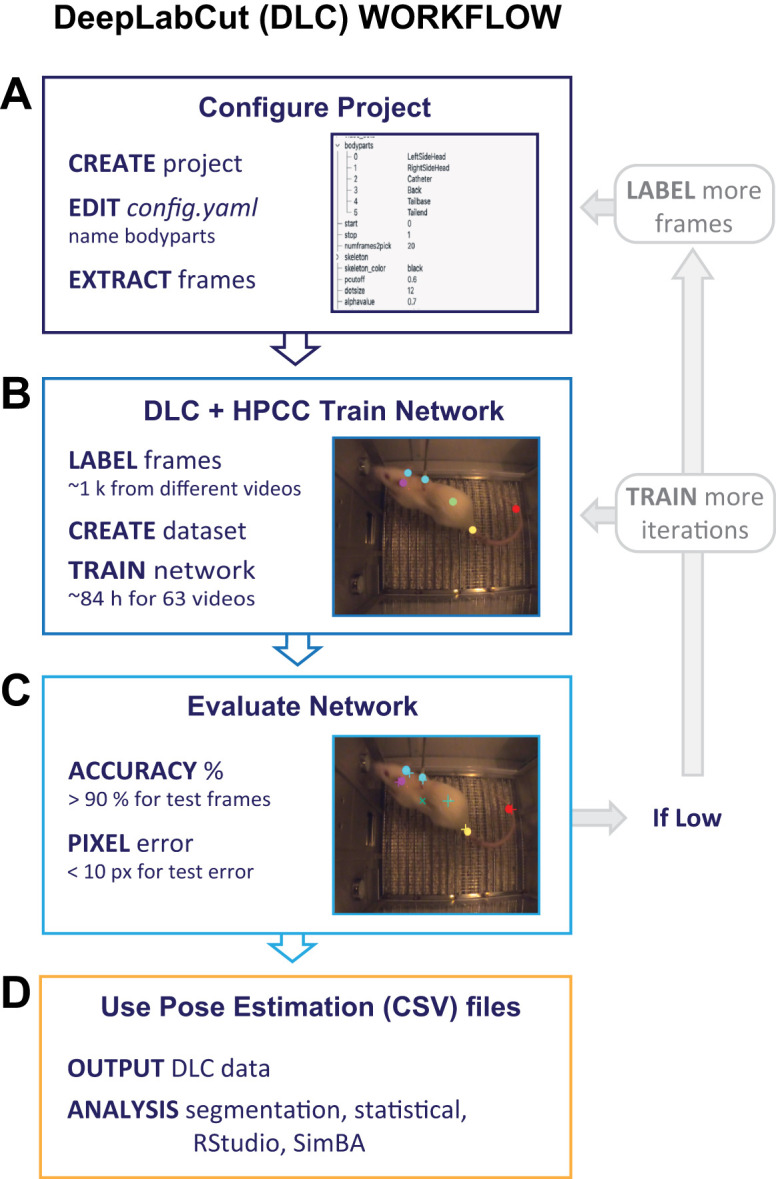
Key steps to acquire pose estimation of lever response behavior: DeepLabCut (DLC) workflow. After acquiring behavioral videos, DLC desktop can be used to ***A*** extract frames with user-defined labels added to various body parts. Labeled frames are then used to ***B*** train the network using MSU's high-performance computer cluster (HPCC). ***C***, The output from DLC network training should be evaluated for test error, training loss, and % accuracy. Depending on the results, more frames can be labeled and/or the network trained for more iterations (gray-shaded text), or the output can proceed to ***D*** segmentation of statistical analysis.

Open-source toolboxes, such as DLC, provide a machine learning approach to predict the spatial positions of body parts during motor behavior (Mathis et al., 2018; Nath et al., 2019; Luxem et al., 2022; Isik and Unal, 2023). These analyses can reveal detailed behavioral information to complement lever response data acquired with Med-PC and can be less time intensive than manual annotation of videos (manual annotation of a 2 h Coc-SA session = minimum of 3 h). We used DLC (version 2.3.8) for body part tracking on a Windows 11 PC. A total of 45 videos with 1,920 × 1,080 px resolution (width × height) were used to extract frames for network training. Key protocol steps in the DLC workflow outlined in Figure 3 include “Labeling videos outside HPCC, Interfacing DLC with HPCC, Training and evaluating the network, and Video analysis.” Briefly, to achieve the best possible model, four different networks were trained following the DLC user guide (Table 3). For the most accurate network, six different body parts were labeled: “LeftSideHead,” “RightSideHead,” “Catheter,” “Back,” “TailBase,” and “TailEnd.” After ≥500 frames were labeled, network training was performed through MSU's HPCC using a Linux/Ubuntu OS. DLC version 2.3.5 was installed on the HPCC using the default environment clone from the GitHub repository. Installation of TensorFlow on MSU's HPCC followed guidelines for Conda version 3 (Anaconda3-2022.05-Linux-x86_64; https://docs.icer.msu.edu/Using_conda), as outlined in the MSU HPCC documentation (see methods below for the TensorFlow installation script). Prior to model training, labeled data were prepared using an interactive session with 100 cores per task, 1 GB of CPU memory, and three AMD21 GPUs, each equipped with 81.92 GB of memory. Model tracking utilized a single 8 GB GPU through an array Slurm batch submission, optimized for nodes with AMD20 V100 GPUs, as Conda had been installed specifically for these nodes.

**Table 3.** Key information for Networks 1–4 trained to track rats during cocaine self-administration

Network	1	2	3	4
Frames in training data set	1,500	417	985	1,100
Training iterations	650,000	850,000	630,000	950,000
DeepLabCut test error with *p* cutoff (pixel)	9.60	6.42	21.99	9.38
Measured accuracy (%)	91.05	98.10	46.61	99.12
Resolution	640 × 480	640 × 480	3,840 × 2,160	1,920 × 1,080

Network 1 used a larger training data set with labels for body parts (left head, right head, catheter, back). Network 2 used a smaller training data set and increased iterations and labels for levers (active, inactive) and body parts (head, back). Network 3 used a higher resolution video and labels for body parts (left head, right head, catheter, back, tail base, tail end) as well as a global scale value of 0.4. Network 4 used a lower resolution than 3. All networks had sufficient training iterations (>200,000) with low training loss. Network 4 resulted in a higher manual evaluation of training accuracy while keeping the video resolution relatively high.

After editing video paths and creating a training data set, the network was trained until pixel loss error was <0.01 or at least 200,000 iterations occurred. Specifically, we used a ResNet-50–based neural network with default parameters for training and a 0.95 train test split. Network evaluation was performed with a *p* cutoff of 0.8 for test error. Accuracy counts were also performed by calculating the % of frames labeled correctly after manually counting incorrectly labeled body parts for frames used during network testing. After the model was deemed fit, 63 videos were then analyzed, and the results were saved as csv files. Detailed protocol steps for the DLC workflow are provided below.

##### Label video frames outside HPCC

This section follows steps A–D outlined in the DLC User Guide with additional information found in the DLC installation guide. The DLC GUI for labeling frames does not work inside the HPCC's interactive desktop; therefore, it is necessary to install DLC on a personal computer (Windows OS recommended) and label it accordingly (Fig. 3*A*).

1. Install DLC using a PC connected to a Wi-Fi network, following guidelines for the GUI installation. General steps for Windows OS are outlined below:
  • Install Anaconda and Git.
  • Download the *.yaml* file from DLC's GitHub repository.
  • Open “Anaconda Prompt” with administrator access. Find the folder containing the .*yaml* file (i.e., enter ***cd Downloads*** into the terminal).
  • Run ***conda env create -f DEEPLABCUT.yaml***.
2. To open the GUI installation of DLC, run ***conda activate DEEPLABCUT*** then enter ***python -m deeplabcut*** in the terminal. Click on “Create a New Project,” type in the desired project and experimenter's name, and select behavioral videos.
3. Define labels. In the “Manage Project” tab, click on “Edit config file,” and add head, catheter, back, and tail labels to the “bodyparts” parameters: ***- LeftSideHead, - RightSideHead, - Catheter, - Back, - TailBase, - TailEnd***.

Note: The code shown above should be written in a list format (box on the left, label index; box on the right, corresponding name). Back refers to the middle point between a rodent's tail base and the catheter.

4. Click on the “Extract Frames” tab to take frames for each video (∼5–10 h). Greater than or equal to 500 frames split across multiple videos of different animals should be extracted (Clemensson et al., 2020).
5. Click on the “Labeling” tab, label select rat body parts from extracted video frames, and then save the data. Skip over any label if a body part is not visible in the frame.

##### Interface DLC with HPCC

Training without access to a GPU can be lengthy (i.e., >72 h); therefore, the use of a GPU via Google Colab or HPCC is recommended. The following steps outline how to install DLC inside HPCC:

1. Access the DLC containers on Docker Hub. On the website, click on “Tags,” and copy the latest version of the pull command. The Docker pull command might look like ***docker pull deeplabcut/deeplabcut:2.3.5-base-cuda11.7.1-cudnn8-runtime-ubuntu20.04-latest***. Docker will be used in Step 4.
2. Set HPCC parameters. Go to ondemand.hpcc.msu.edu, and click on “Interactive apps” and then “Interactive desktop.” Adjust or use the parameters suggested below, and then click on “Launch” at the bottom of the page.
  • Number of hours: 4 for small jobs or 60 for training
  • Number of cores per task: 100
  • Amount of memory per CPU core (RAM): 1 GB
  • Node type: amd21
  • Number of GPUs: 3
3. Once the session loads, click on “Launch Interactive Desktop.” Go to the top left corner and click on “Applications>System Tools>Terminal.” In the terminal, access the desired folder from folders in your user account.
4. Run ***singularity build –sandbox ./Downloads/DLC<name_of_container>*** by inserting the Docker pull command that was copied in Step 1 in the container name field.
  • Downloading the Docker container of DLC requires using Singularity. However, Singularity's containers are usually read-only. To override this, a sandbox (a writable directory) is created.
5. Run the command shown to access the Docker image with DLC installed in it: ***singularity run –writable –nv ∼/Downloads/DLC***.
6. DLC should now be properly installed. In the terminal, run ***python*** and then ***import deeplabcut*** to get started.

Note: Once DLC is installed, open a new interactive desktop session, use the terminal to access the directory where DLC was downloaded, repeat Steps 5 and 6.

##### Train the network through HPCC

1. Create a new project. In the Interactive Desktop terminal, run ***deeplabcut.create_new_project(<name of the project>, <name of the experimenter>, [<name of video directory>])***.
2. Configure the Project. To access the *config.yaml* file, go to the HPCC home directory.
  • Find the *config.yaml* file in your project folder and add the following (in the format of a list) to the “bodyparts” parameter. (as outlined in the GUI protocol): ***- LeftHead, - RightHead, - Catheter, - Back, - TailBase, - TailEnd***.
  • Press “Ctrl+S” to save the changes made.
3. Add labeled data. Copy all folders with labeled data for each video, find the HPCC's project directory, and paste it into the “labeled-data” folder.
4. Double-check the *config.yaml* file. Under the parameter **video_sets** in the *config.yaml* file, make sure that all paths for the corresponding videos are present, and specify where to crop the video.
  • For example, the following path points to a video of a rat in the HPCC's project directory: ***/mnt/home/user/Downloads/Videos/P8,2021-12-21,23-50-18.mp4 crop 0, 640, 0, 480***.
5. Create dataset. In the terminal, run ***deeplabcut.train_dataset(<config_path>)***.
  • ***<config_path>*** refers to the folder path where the *config.yaml* file is.
  • Running the code above will create a file called *pose_cfg.yaml*. Look for the file inside the “train” subdirectory found in the “dlc-models” folder of the project directory. If not found, training should not be started.
  Note: Run ***deeplabcut.check_labels(<config_path>)*** and check the “labeled_data” folder to make sure that DLC recognizes all labeled videos.
6. Train the network (∼5–8 h). Run ***deeplabcut.train_network(<config_path>)***. Once the training loss drops to below 0.01 or at least 200,000 iterations (visible in the terminal), end the training by typing “Ctrl+C.”

Note: Training can be restarted from a checkpoint. The directory where the *pose_cfg.yaml* file is found saves checkpoints of your training. See the DLC guideline for more information.

##### Evaluate the network

Run ***deeplabcut.evaluate_network(config_path)***. This will output a csv file under the “evaluation_results” directory (Fig. 3*C*). The csv file will contain a test error value (the likelihood that the network will incorrectly label a body part, px). It is recommended that this value is below 10.0.

##### Video analysis

Using a set of new videos, run ***deeplabcut.analyze_videos(<config_path>, [<path_for_video>], save_as_csv=True)***. See the example in the GitHub repository. The video path can be inserted as shown in Step 4. This will output csv files with prediction values for each video, which can be used for creating plots using programming languages like RStudio, Python, and others (Fig. 3*D*).

#### Protocol for analysis of pose estimation output using SimBA (key steps)

**Figure 4.**
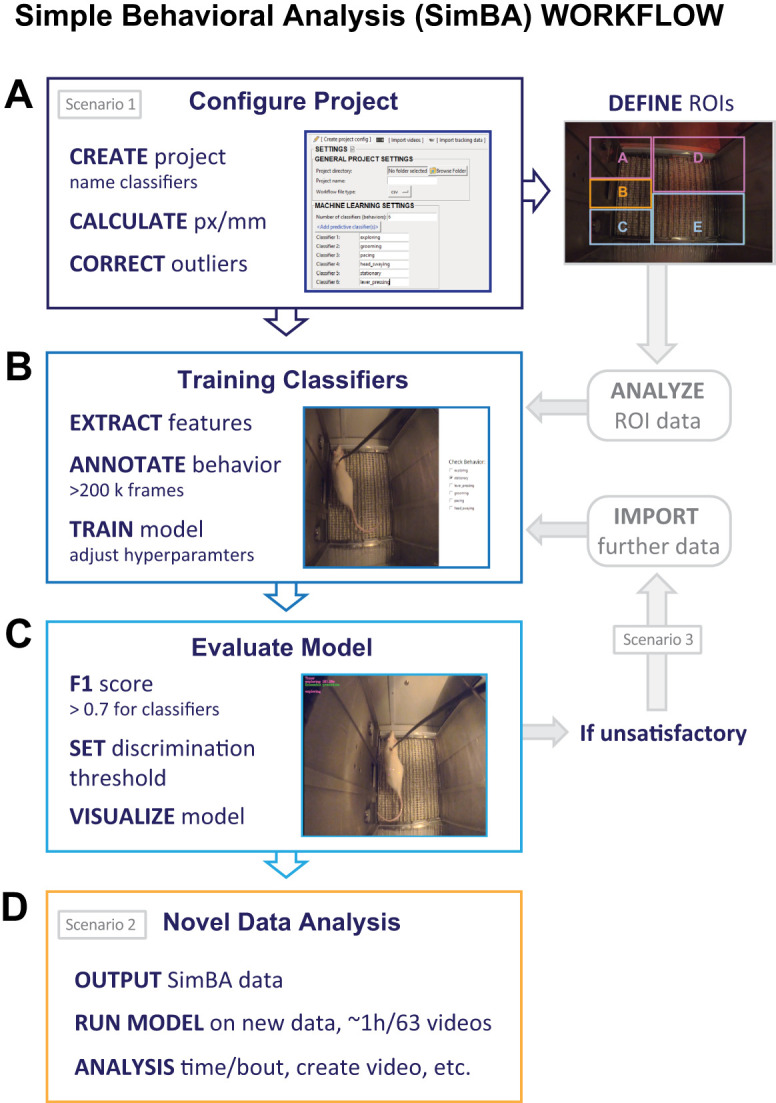
Key steps to acquire region of interest and predictive classifier data: Simple Behavioral Analysis (SimBA) workflow. Pose estimation outputs from DeepLabCut (DLC) can be transferred into SimBA to ***A*** create predictive classifiers and correct outliers based on movement and location (workflow Scenario 1). Regions of interest (ROIs) can then be created with the active lever, Quadrant A; area behind the active lever, Quadrant D; area containing nose poke receptacle, Quadrant B; inactive lever, Quadrant C; and area behind the inactive lever, Quadrant E. Model training can then be performed ***B*** after feature extraction, and >200,000 frames should be annotated with behaviors of interest. ***C***, The models trained can be evaluated using their *F*_1_ score and can be considered a good fit provided visualization of predictive classifiers demonstrate accurate tracking. If necessary, more frames can be labeled (workflow Scenario 3, gray-shaded text) or the output can proceed to ***D*** running the model on new videos (workflow Scenario 2) which can be used for subsequent classifier analysis.

To further explore distinct behaviors in each training phase, we chose to use a machine learning approach to analyze time spent in regions of interest (ROIs) within the operant chamber and generate predictive classifiers of specific behaviors. To accomplish this goal, we used the open-source pipeline SimBA (Nilsson et al., 2020; Goodwin et al., 2024) version 1.94.6 on a Windows 11 PC using Python 3.6. All steps in this section of the protocol were implemented with the Scenario tutorials listed in the SimBAGitHub documentation. Key protocol steps for SimBA workflow outlined in Figure 4 include “Defining ROIs from DLC outputs, Annotating and training classifiers, Model evaluation, and Analyzing SimBA outputs.” Specifically, we recommend following the steps in Scenario 1 for creating a user-defined pose configuration, outlier correction, ROI analysis, behavioral annotation, model analysis, and evaluation. After classifier models were created, we followed steps in Scenario 3 to improve models until they were deemed fit for accurate classification and DLC processed videos could be used for data analysis in Scenario 2.

##### Define and generate ROI from DLC pose estimation outputs

Of the 63 videos analyzed through DLC, 8 videos were imported into the project folder with their corresponding DLC csv files. Pixels/millimeter values for each video (∼6.0 px/mm for all) were calculated by manually measuring the width of the operant box chamber floor and measuring the same distance through the SimBA GUI. Outlier corrections were performed, as recommended in the SimBA tutorial, using the catheter and tail base body parts, a location criterion of 1.5, and a movement criterion of 1.0, (Fig. 4*A*). To determine the location of key rat body parts during behavior, we followed the SimBA tutorial for ROI analysis using the same videos imported for behavioral predictions. For each video, five quadrants were drawn using the SimBA toolkit (Fig. 4*A*, define ROIs). The five quadrants were based on key areas of the operant chamber: A, quadrant containing the active lever; D, quadrant behind the active lever; B, nose poke; C, quadrant containing the inactive lever; and E, quadrant behind the inactive lever. ROI data were generated for the left head, right head, and catheter body parts using a probability threshold of 0.8. The results were csv files containing statistics that describe behavior classifications, including bout count, mean event duration (in seconds), and total event duration (in seconds) for each ROI. To examine orientation to the active lever, wind rose plots were generated by calculating the angle between two vectors: one from the center of the head to the catheter points and another from the active lever to the center head. We divided these angles into 30° bins and then recorded the frequency of each orientation throughout each session, as shown in Figure 6*E*.

##### Annotate and train classifiers

A total of 239,130 frames were annotated for the presence or absence of the following behaviors (Table 4): exploring, grooming, and pacing. We chose these behaviors based on the SimBA body label tutorial, our observations from manual scoring of 63 videos, and previous literature (Sams-Dodd, 1995; Nilsson et al., 2020; Goodwin et al., 2024; Popik et al., 2024). Of the annotated videos, 15–30% included frames where the behavior was present. For model training, we used the default values in the hyperparameter and evaluation settings provided in the Scenario 1 metadata file with the exception of using an entropy criterion, choosing frames for the type of train test split, and the generation of classification reports (Fig. 4*B*).

**Table 4.** Description of characteristics used to annotate behaviors for training predictive classifiers in SimBA

Classifier	Description	Start frame	End frame
**Exploring** 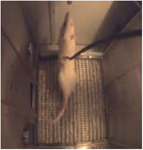	Curiosity in surroundings Poking head in food dispenser Climbing on walls Biting catheter line	Rat lifts its front paws, starts moving head toward the environment Pokes nose inside food dispenser	For climbing, catheter nibbling, frame before both paws are completely still on the floor or rat stops moving body or head Frame before rat consistently starts moving quickly without direction Nose poking: last frame before the nose is withdrawn from inside the box
**Grooming** 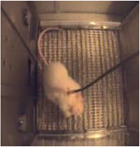	Rat is curled in Rapid motions as rat brushes, cleans itself Paws over the head, scratches stomach in rapid motion with the legs Teeth nibbling on the back or stomach	Rat's head is not laid out flat and body contracts into itself Exception: when rat's head is laid out flat but one paw is lifted to scratch body. Rat will often stay still for a few seconds before it starts grooming	Frame before a shudder (rat's torso shakes abruptly) or sudden cease of motion and body is laid out flat Frame before the rat quickly moves to another part of the box and stops brushing, scratching, or nibbling
**Pacing** 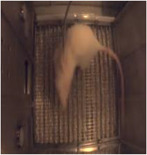	Rat moves quickly inside the box Motion can be circular arriving at the same point or linear without exploration behaviors (such as climbing/nose poking) Characterized by rat moving back and forth from one part of the box to another	Initial frame will often be blurry Rat will quickly change position from one place to another	Any frame where rat remains stationary or begins another behavior such as exploring Last frame before the end of a sudden movement (often a blurry image)

##### Evaluate model

We examined *F*_1_ scores to determine which model to use to evaluate annotated videos. The *F*_1_ score is comprised of the harmonic means of both precision and recall measures. Precision is the ratio of correctly predicted positive observations to the total predicted positives (true and false positives). Recall is the ratio of correctly predicted positive observations to all observations in the actual class (true positives and false negatives). An *F*_1_ score of 1 means accurate model performance while a value of 0 means inaccurate model performance. The models that yielded the highest *F*_1_ score were chosen for evaluation using one of the eight previously annotated videos. Multiple videos were created using different discrimination thresholds, and the value (approximately 0.8 for all models) that yielded the most accurate visualization of behavior detection was used for postevaluation. During evaluation of each classifier, a minimum bout length of 180 ms (equivalent to 4 frames in a 24 frames/s video) was used to smooth out behavioral predictions (Fig. 4*C*).

### Novel data analysis

After models were deemed fit for classification, 63 videos including their pose estimation csv files were imported into a new project folder following the instructions from Scenario 2. Videos were then analyzed using predictive classifiers (exploring, grooming, pacing), and generated csv files were used to calculate the frequency of each behavior over the different phases of self-administration behavior (Fig. 4*D*).

### Code accessibility

The code related to video acquisition is freely available online at https://github.com/fmaureira/ArguelloALab. The code is available as extended data.

### Statistical analysis

To examine for differences between responses on the active or inactive lever or time spent in active (A and D) or inactive (C and E) quadrants, one-way repeated-measures ANOVAs were conducted, with follow-up Tukey's post hoc comparisons when significant main effects were observed. To examine for differences between responses on the active and inactive lever or time spent in the active (A and D) and inactive (C and E) quadrants, paired *t* tests were conducted for the average of the last three sessions of Coc-SA. Alpha was set at *p* < 0.05.

## Results

### Video acquisition

Obtaining behavioral videos with RasPi or GoPro cameras was relatively straightforward. The RasPi setup involved steps to install RasPi microcomputers, program RasPi cameras, and the Termius web application and SSH client to remotely access the RasPi camera. The GoPro camera setup was user-friendly, requiring less prerecording installation, but initiation of videos cannot be controlled with a graphical user interface (Fig. 2). Key parameters in video acquisition included: determining the appropriate resolution and frames per second to obtain high-quality videos that do not require extensive processing time during DLC network training. The GoPro Quik app can be used to combine segmented videos, but other processing software or apps are available including Ffmpeg or Adobe Premiere Rush. We suggest daily extraction and organization of videos to ensure accurate field of view, length, date, and time stamps of videos are collected. Errors in any of these parameters will result in truncated videos and complicate pose estimation data analysis.

### Obtaining pose estimation outputs from DLC

A primary goal of our study was to obtain pose estimation outputs from behavior videos and DLC network training using MSU's HPCC (Fig. 3, “DLC workflow”). We first labeled specific frames extracted from videos by DLC (Fig. 3*A*). Based on previous literature, we labeled several rat body parts including the left head, right head, base of the jugular catheter, base of the tail, and end of the tail (Centanni and Smith, 2023). We found that labeling frames using DLC was relatively straightforward but considerable troubleshooting was needed to install all necessary packages for DLC to run properly. Extensive time will initially be needed to evaluate the network and determine the appropriate number of frames needed to sample a wide range of behaviors in all phases of training. This process will become more efficient once the model is trained (Fig. 3*B,C*).

We next trained the DLC network on labeled frames (Fig. 3*B*). DLC uses CNN to determine the spatial position of a labeled body part (Mathis et al., 2018; Nath et al., 2019). We utilized MSU's HPCC to allow for faster training times with multiple GPUs. Networks 1 and 2 were trained with lower-resolution RasPi recordings (640 × 480 px). We labeled 1,500 frames, ran the network for 650,000 iterations (∼12 h of training time), and obtained the following values for Network 1 training: test error of 9.60 (with *p* cutoff = 0.8), meaning there was a 9.6% probability that the predicted labels from the network were considered unreliable (Table 3). The training pixel loss error, which measures the mean average Euclidean error between manual labels and predicted labels was <0.01 (Mathis et al., 2018; Nath et al., 2019). The measured % accuracy was 91.05, calculated by manually counting incorrect DLC labels for each body part and dividing them by the total amount of labels. We observed that DLC performed poorly on areas that might not be captured on each frame (e.g., tail base). During the process of calculating % accuracy, we also found that several videos did not capture the entire operant field, and thus we removed these videos and then relabeled frames (Fig. 3*B*, gray-shaded area). Due to the reduced number of videos, for Network 2 we labeled 417 frames (with body parts of center head and back) and trained for 850,000 iterations (∼18 h of training time). Overall, the values from Network 2 training improved: test error, 6.42 px; training loss, <0.01; and % accuracy, 98.10 (Table 3).

To improve video collection (less variable field of views and improved resolution), Network 3 was trained with videos obtained using GoPro cameras from Cohort 2. For Network 3, we labeled 985 frames (with body parts of the left head, right head, catheter, back, base of tail, and end of tail), trained for 630,000 iterations, and reduced the global scale hyperparameter (found inside *pose_cfg.yaml*) to adjust for larger resolution videos (3,840 × 2,160 px) and avoid frame reading errors. Results were unsatisfactory, with test error, 21.99 px; training loss, 0.02; and % accuracy, 46.61, likely due to the error associated with large video resolution. Network 4 was then trained using similar body part labels, reduced video resolution (1,920 × 1,080 px), and a default global scale value of 0.8. Evaluation values improved significantly with test error, 9.38 px; training loss, <0.01; and % accuracy, 99.12 (Table 3).

For DLC HPCC network training (Fig. 3*B*), we spent significant time troubleshooting steps related to the “create training dataset”: in particular, enabling write access to Docker images inside HPCC and reformatting files with labeled data. To avoid extensive troubleshooting on this step, we recommend installing DLC on HPCC using Singularity to read Docker images, determine whether DLC recognizes labeled data using the check annotated frames function provided in the user guide manual, and maintain consistent project name configurations, when DLC projects are created on different PCs, to avoid data merge conflicts.

### Analysis of pose estimation output using SimBA

**Figure 5.**
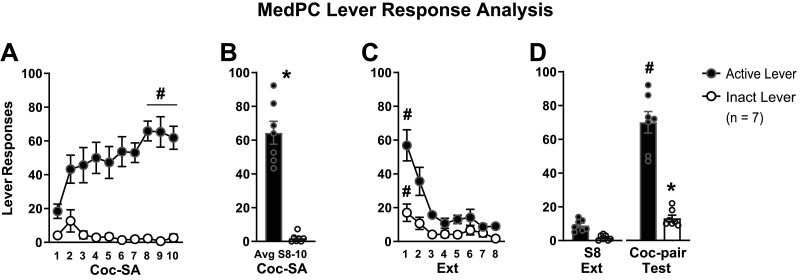
Med-PC lever response analysis. Rats were trained to respond for cocaine rewards. An active lever response resulted in cocaine infusion, whereas an inactive response resulted in no consequences. ***A***, Over the 10 sessions of cocaine self-administration (Coc-SA) training, active lever responses increased (S1 < S8–10, ^#^*p* < 0.01), in contrast to inactive responses which remained low throughout (S1 = S8–10). ***B***, The mean number of responses on the last three sessions of Coc-SA was significantly higher for the active versus inactive lever, *p < 0.01. ***C***, Over the eight sessions of extinction (Ext) training, active and inactive responses decreased (S1 < S8, ^#^*p* < 0.01). ***D***, During the Relapse Test, active lever responses were higher in the cocaine-paired (Coc-pair) context compared with the last session of Ext (Ext S8 < Coc-pair, ^#^*p* < 0.01), and Relapse Test active responses were higher than inactive (*p < 0.01).

**Figure 6.**
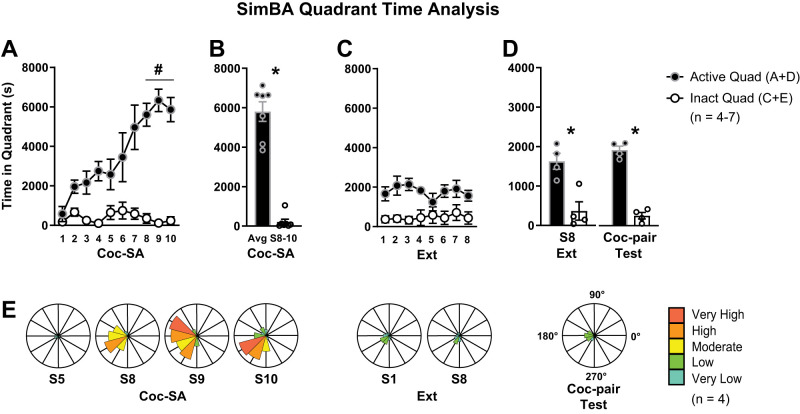
SimBA quadrant time analysis. Rats were trained to respond for cocaine rewards. An active lever response resulted in cocaine infusion, whereas an inactive response resulted in no consequences. ***A***, Over the 10 sessions of Coc-SA training, time spent in the active quadrant (A and D) increased (S5 < S8–10, ^#^*p* < 0.05), in contrast to time spent in the inactive quadrant (C and E), which remained low (S5 = S8–10). ***B***, The mean time spent in quadrants on the last three sessions of Coc-SA was significantly higher in the active versus inactive quadrant, *p < 0.01. ***C***, Over the eight sessions of extinction (Ext) training, time spent in both the active and inactive quadrants remained stable. ***D***, During the Relapse Test, time spent in the active and inactive quadrants was similar in the cocaine-paired (Coc-pair) context compared with the last session of Ext. Active responses were higher than inactive during the last session of Ext and the Coc-pair Test (*p < 0.01). ***E***, Wind rose plots depicting the orientation of rats to the active lever during Coc-SA (S5, 8–10), Ext (S1, 8), and Relapse Test. Plots were generated by calculating the angle between two vectors: one from the center head point to the catheter point and another from the active lever to the center head point. Angles were divided into 30° bins, with 0° notating orientation to the active lever. Frequency of orientation throughout each session: very high (dark orange) to very low (teal).

A second goal of the current study was to use pose estimation outputs from DLC to examine whether time spent in specific quadrants correlated with lever responding behavior. While several post-training software are available for video analysis, we chose SimBA because it integrates DLC outputs in its workflow. We first analyzed standard lever responses from Med-PC (Fig. 5), followed by a SimBA-mediated analysis of DLC outputs (Fig. 6).

#### Med-PC lever response analysis

Across Coc-SA training sessions, we found that rats responded more on the active (black circles) compared with the inactive (white circles) lever (Fig. 5*A*). 1 × 10 ANOVAs for active or inactive lever responses across Coc-SA sessions revealed significant effects (active, *F*_1, 9 _= 5.080, *p* < 0.001; inactive, *F*_1, 9 _= 2.081, *p* = 0.048). Tukey's post hoc comparisons revealed higher active responses on the last three sessions of Coc-SA compared with the first session (S1 < S8–10; *p* < 0.01), with no differences in inactive responses between the first and last three sessions (S1 = S8–10). The average lever responses on the last three sessions of Coc-SA revealed that rats responded significantly more on the active lever than the inactive lever (Fig. 5*B*, *t*_6 _= 9.23, *p* < 0.0001).

During Ext training, rats reduced lever responses by the final Ext session (Fig. 5*C*). 1 × 8 ANOVAs for active and inactive lever responses across Ext sessions revealed significant effects (active, *F*_1, 7 _= 13.822, *p* < 0.001; inactive, *F*_1, 7 _= 5.894, *p* < 0.001). Tukey's post hoc comparisons revealed higher responses on the first Ext session compared with the last session for both levers (active and inactive, S1 > S8, *p* < 0.01). During the Relapse Test, responses were significantly higher in the previous Coc-pair context (Fig. 5*D*). An ANOVA for active and inactive responses revealed significant effects (*F*_1, 3 _= 79.79, *p* < 0.001). Tukey's post hoc comparisons revealed higher responses on the active lever during the Relapse Test in the Coc-pair context compared with the last session in the Ext context (Ext < Coc-pair, *p* < 0.01), with no differences on the inactive lever. In addition, responses were higher on the active versus inactive lever during the Relapse Test (active > inactive, *p* < 0.01).

#### SimBA quadrant time analysis

The time spent in the active quadrant was calculated as the sum of the time spent in Quadrants A and D, and time spent in the inactive quadrant was calculated as the sum of time spent in Quadrants C and E (Fig. 4, ROIs). We combined time spent in Quadrants A and D given our observations during manual video annotation of rats moving between the active lever and quadrant behind the active lever during Coc-SA. For our first cohort, videos were collected only from Coc-SA, and we labeled body parts of the center head and catheter. For a second cohort of rats, videos were collected from all behavioral phases (Coc-SA, Ext, and Test), and we labeled body parts of the catheter, right head, and left head. The overall pattern of time spent in quadrants determined by catheter, right, or left head ROIs was similar in each behavioral phase. Therefore, we calculated a center head value for time spent in quadrants to allow for data comparison between both cohorts of rats (presented in Fig. 6). Across Coc-SA training sessions, we found that rats spent more time in active quadrants (A and D; Fig. 6*A*, black circles) than in inactive quadrants (C and E; Fig. 6*A*, white circles). 1 × 6 ANOVAs for time spent in active or inactive quadrants across Coc-SA sessions revealed significant effects (active, *F*_1, 5 _= 7.845, *p* < 0.001; inactive, *F*_1, 5 _= 4.245, *p* = 0.005). Tukey's post hoc comparisons revealed more time spent in active quadrants on the last three sessions of Coc-SA compared with the fifth session (S5 < S8–10; *p* < 0.05), with no differences in inactive responses between the fifth and last three sessions (S5 = S8–10). The average time spent in active (A and D) versus inactive (C and E) quadrants on the last three sessions of Coc-SA revealed that rats spent significantly more time in the active versus inactive quadrants (Fig. 6*B*, *t*_6 _= 8.43, *p* = 0.0002).

During Ext training, rats spent a similar amount of time in the active and inactive quadrants between the first and final Ext sessions (Fig. 6*C*). 1 × 8 ANOVAs for time spent in the active and inactive quadrants across Ext sessions revealed no significant effects (active, *F*_1, 7 _= 0.942, *p* = 0.496; inactive, *F*_1, 7 _= 0.393, *p* = 0.896). During the Relapse Test, time spent in the active quadrant was similar during Ext and the Coc-pair context (Fig. 6*D*). An ANOVA for time in the active and inactive quadrants revealed significant effects (*F*_1, 3 _= 21.54, *p* < 0.001). Tukey's post hoc comparisons did not reveal significant differences in lever responses in the previous Coc-pair context compared with the last session in the Ext context (Fig. 6*D*). However, time spent in the active quadrant was higher than in the inactive quadrant during the last Ext session and Relapse Test (active > inactive, *p* < 0.01). The data suggest that machine learning generated time spent in the active and inactive quadrants paralleled lever responses during Coc-SA. In contrast, time spent in the active and inactive quadrants did not parallel lever responses during the Ext training and Coc-pair Tests (both phases during which drug rewards are not present).

To explore non lever-dependent behaviors further, we examined the orientation of rats to the active lever during Coc-SA (S5, 8–10), Ext (S1, 8), and Relapse Test. We generated wind rose plots by calculating the angle between two vectors: one from the center head point to the catheter point and another from the active lever to the center head point. We divided these angles into 30° bins, with 0° notating orientation to the active lever, and then recorded the frequency of each orientation throughout each session (Fig. 6*E*). During the later phases of Coc-SA (S8–10), we observed a higher frequency of orientations between 135° and 250° compared with S5. In contrast, no specific orientation pattern was noted during Ext or the Coc-pair Test. From our manual observations, we noted that during the final session of Coc-SA, the rats tended to orient away from the active lever, facing either the wall opposite the lever (Quadrant D) or along the wall between Quadrants A and D. In some instances, rats were huddled below the active lever with their head oriented away from the lever.

### Generating predictive classifiers of behaviors using SimBA

**Figure 7.**
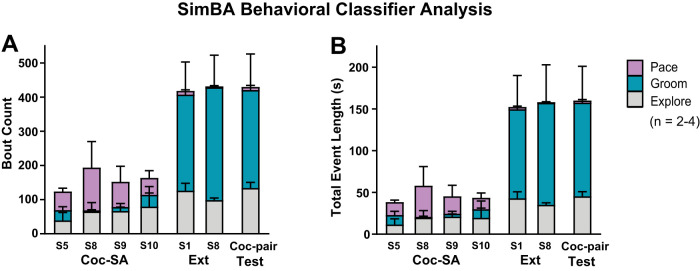
SimBA behavioral classifier analysis. Predictive classifier analysis for ***A*** Bout count and ***B*** Total bout length (in seconds) for exploration, grooming, and pacing behaviors. Exploration was present in all phases of behavior (gray-shaded), but pacing was primarily present during Coc-SA (pink-shaded), with more grooming behaviors present during Ext and Relapse Test phases (teal-shaded). Definitions of exploration, grooming, and pacing behaviors are noted in Table 4.

**Figure 8.**
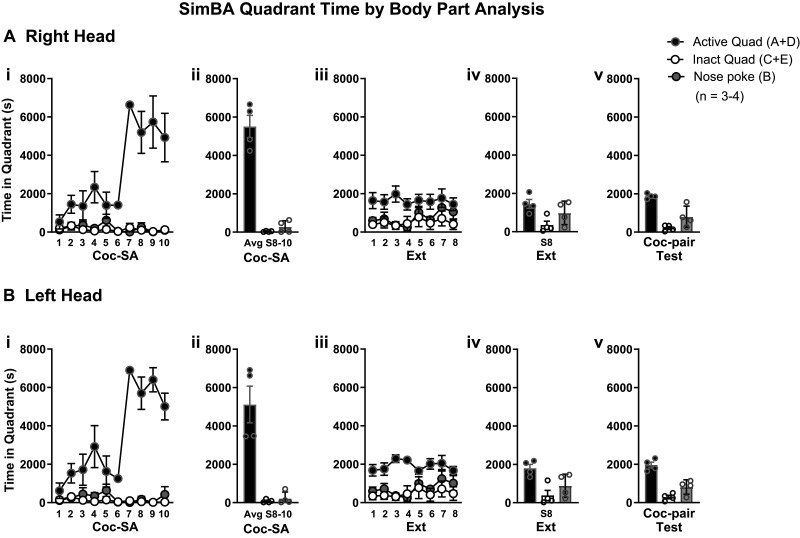
SimBA quadrant time analysis. Parallel Figure 6 Results from time in operant quadrants generated with SimBA for the ***A*** right head and ***B*** left head body parts. Rats were trained to respond for cocaine rewards. An active lever response resulted in cocaine infusion, whereas an inactive resulted in no consequences. Qualitative presentation of time spent in the active quadrant (A and D), compared with the inactive (C and E) or nose poke ***B*** quadrants during ***A***, ***Bi,ii*** cocaine self-administration (Coc-SA) phase, ***A***, ***Biii*** extinction (Ext) training, ***A***, ***B,iv*** the last session of Ext, and ***A***, ***B,v*** during the Relapse Test in the cocaine-paired (Coc-pair) context.

A final goal of the current study was to determine if pose estimation data could be analyzed using another supervised machine learning pipeline to generate predictive classifiers of behavior. We therefore input the pose estimation data from DLC network training into the SimBA pipeline to create predictive classifiers to reveal patterns of behavior associated with specific phases of behavioral training, with a focus on non lever response behaviors. We qualitatively examined Bout Counts and Total Bout Length (in seconds) for exploring, grooming, and pacing behaviors (Table 4) for Coc-SA (S5, 8–10), Ext (S1, 8), and Test from calculated center head values (Fig. 7*A,B*, see qualitative right and left ROI plots in Fig. 8*A,B*). We found that exploration was present in all behavioral phases (grey-shaded areas). Grooming was observed during Coc-SA, but was surprisingly more dominant in phases without drug reward (teal-shaded area, Ext and Test). Pacing was primarily observed during Coc-SA, with minimal presence during Ext and Test phases (pink-shaded areas). 

## Discussion

In the current study, we provide protocols to (1) collect videos from operant self-administration using Raspberry Pi (RasPi) or GoPro cameras, (2) extract pose estimation data using the supervised machine learning software DeepLabCut (DLC), (3) compare standard Med-PC lever response versus quadrant time data generated from DLC pose estimation, and (4) generate predictive behavioral classifiers generated from Simple Behavioral Analysis (SimBA).

### Video recording

Overall, the use of the RasPi and GoPro cameras allowed for the collection of high-quality videos in .mp4 format, for easy visualization and contrast adjustment if needed. The current methods outlined the installation and use of the Termius application, which allows the user to easily control and initiate RasPi video recordings. We also provided a second method to collect videos using GoPro cameras, which required fewer prerecording installation steps, but did not allow for video control with a computer interface. The current methods can be readily interfaced with existing real-time backup methods to scale up video collection in the future (Goodwin et al., 2022; Centanni and Smith, 2023; Isik and Unal, 2023). An important consideration for future studies is the efficient organization and notation of any truncated behavioral videos once they are collected to facilitate subsequent pose estimation data analysis.

### Pose estimation analysis using DLC

We acquired videos of operant behavior from two cohorts of rats: one cohort consisted of cocaine self-administration (Coc-SA) behavioral training, and the second cohort included all behavioral phases—Coc-SA, extinction (Ext), and Relapse Test. To obtain pose estimations from videos, we chose a supervised machine learning model that utilizes pretrained neural networks (Luxem et al., 2023). While several supervised and unsupervised models are available, we used DLC due to the extensive troubleshooting forum and resources available through GitHub and the wide use of DLC pose estimation outputs with other behavior detection software (e.g., BehaviorDEPOT, VAME, and SimBA). We encountered significant challenges in setting up DLC and its associated packages, particularly when running the software without GPU access, which could be an initial hurdle for new users. To minimize installation issues, we reduced video resolution and file size, used a Windows OS, labeled data through the GUI, and utilized the DLC Docker container installation. This Docker setup, which includes all necessary dependencies, simplified installation and facilitated access to a GPU within MSU's HPCC (https://icer.msu.edu/web-portal-hpcc-resources).

Once all critical software was installed, labeling DLC video frames was relatively straightforward and efficient (0.25 h/video). We first chose to label four rat body parts (left head, right head, catheter, and back), which resulted in approximately 35 frames/video, for a total of >700 labeled frames (Nath et al., 2019; Clemensson et al., 2020). We then used MSU's HPCC to train the network. Table 3 shows the important values which allowed us to evaluate network performance: test error with *p* cutoff, the probability that predicted labels from the network were considered unreliable; training loss, the mean average Euclidean error between manual labels and predicted labels; and % accuracy, the number of incorrect DLC labels for each body part divided by the total number of labels. For Network 1 training, we observed low % accuracy likely due to video quality issues including shifted fields of view, differing zoom angles, truncated length, or low contrast. Therefore, we adjusted parameters to improve the network evaluation by removing videos that did not capture the entire operant field, labeled more frames, and increased the number of training iterations (650,000 vs 850,000). Overall, Network 2 training data values improved, with similar % accuracy (95%) for both head and back labels.

We subsequently trained two networks which included videos from a second cohort of rats that went through Coc-SA, Ext, and Relapse Test phases of operant behavior and collected 160,000 frames/video for a total of 1.5 million frames (Goodwin et al., 2022, 2024; Luxem et al., 2023). For Network 3, we labeled six rat body parts (left head, right head, catheter, back, base of tail, end of tail) which resulted in 985 labeled frames and trained for 630,000 iterations. Network 3 resulted in poor performance and frame reading errors, likely associated with a video resolution that was too high (3,840 × 2,160 px, default GoPro setting). Therefore, we reduced the resolution of videos (1,920 x 1,080 px), and Network 4 was trained using similar body part labels, and this resulted in significantly improved network performance (test error with *p* cutoff = 9.38 px; training loss, <0.01; % accuracy, 99.12; Table 3).

### Pose estimation and predictive classifier analysis of behavior using DLC and SimBA

With the pose estimation outputs from DLC, we then utilized the SimBA pipeline to generate regions of interest (ROIs) to analyze time spent in specific quadrants of the operant box. We chose to calculate the active lever quadrant as the sum of the time spent in the column associated with the active lever (Quadrants A and D) and the inactive lever quadrant as the sum of the time spent in the column associated with the inactive lever (Quadrants C and E, Fig. 4). We reasoned to combine the time spent in quadrants based on our manual observations of rats moving between Quadrants A and D during Coc-SA. With SimBA-generated ROIs, we observed similar patterns of responses and quadrant time during the Coc-SA phase. Active responses and quadrant time both increased during the last three Coc-SA sessions, whereas inactive responses and quadrant time remained low. In addition, the average active responses and quadrant time on the last three Coc-SA sessions were higher than inactive measures. In contrast, we observed different patterns of active responses and quadrant measures during the Ext and Relapse Test phases. During Ext training, both responses were significantly higher on the first Ext session compared with the last, whereas both quadrant times remained similar from the first to last session. During the Relapse Test, active responses were significantly higher in the Coc-pair context compared with the last Ext session, with no differences in active quadrant times. Notably, the Relapse Test is the first time when rats experience extinction in the Coc-pair context, suggesting that the current machine learning parameters may lack sensitivity to detect the initial increase in nonrewarded lever responses in both contexts.

We also examined the orientation of rats to the active lever to explore if this parameter would correlate with increased active responses or quadrant time during the Relapse Test. We found that rats spent most of their time oriented toward the active quadrants (A and D), but rats were most likely to be facing the wall opposite the active lever (Quadrant D). Similar to quadrant time measures, we did not observe specific orientation patterns during Ext or Relapse Test phases. It is possible that the orientation of the rats was not directed to the active lever given that an explicit cue light was never paired with infusions of cocaine (given our use of a contextual Coc-SA procedure). Our observations of behavior during these phases show rats facing the opposite direction of the active lever in between infusions with potential left-to-right movements or swaying of the head, indicative of stereotypy. Ongoing studies in the lab aim to label eight rat body parts in DLC which will allow us to use SimBA-validated models that calculate more features. This type of data will help us identify more behaviors and more accurately measure orientation. Nevertheless, this data analysis shows the types of rich datasets that can be obtained from machine learning analysis.

We also utilized pose estimation outputs to generate predictive classifiers for exploring, grooming, and pacing behaviors. Notably, pacing behaviors were pronounced during Coc-SA, but not during Ext or Relapse Test phases, whereas bouts of grooming behaviors were dominant during Ext and Relapse Test (both phases when the drug was not on board). Exploration behaviors remained consistent across all phases of behavior. While minimal, it does appear that pacing behaviors are present during the first Ext session and during the Relapse Test (which is the first extinction session in the Coc-pair context), similar to quadrant time analysis. Future directions may focus on analysis of classifiers related to pacing to reveal distinct behaviors that are dominant in Ext versus Relapse Test, examining specific behaviors that occur in close proximity to either infusions/active responses and including a larger range of predictive classifiers to find a set that distinguishes between these two phases.

## Limitations and Conclusions

Overall, our data suggest that SimBA can be used to detect differences in time spent in operant quadrants and generate behavioral classifications that are associated with behaviors during distinct phases of Coc-SA (Graving et al., 2019; Goodwin et al., 2024; Pereira et al., 2022). There are limitations to the current study—the predictive classifier analysis across all behavioral phases was limited to a second cohort of rats, and analysis was restricted to three types of behaviors: exploration, grooming, and pacing. Future studies should examine a larger suite of behaviors related to grooming, exploration, pacing, and head swaying. While we found a unique distribution of pacing behaviors during Coc-SA versus Ext, a further analysis between early versus late Coc-SA training was not possible given that 1 h of video (a total of 2 h) was truncated during the Coc-SA S1–4. In addition, we found missing SimBA-generated classifier values which were not restricted to a single rat. For example, one rat had missing values for pacing during Coc-SA S5 whereas another had missing values for exploration during Coc-SA S10. Perhaps SimBA did not identify these behaviors, DLC had trouble tracking these behaviors, or classification thresholds were set too high. Future studies may try to resolve these issues with larger sample sizes and varying the thresholds for each behavior. SimBA behavioral classifiers have been validated using eight rat body part labels during social interaction between two rats but have not been extensively validated for single-rat operant Coc-SA. Future experiments will focus on labeling up to eight rat body parts to obtain a more comprehensive set of features that can be used for model training (Goodwin et al., 2024). Previous studies have demonstrated the influence of estrous cycling on drug-taking and drug-seeking behaviors (Lynch et al., 2001; Roth et al., 2004; Jackson et al., 2006; Kerstetter et al., 2008; Nicolas et al., 2019). While the current study focused on behavioral profile analysis in male rats, future studies will include female rats to assess sex-specific differences in behavior.

Despite these limitations, the current study showed a proof-of-principle methodology to obtain rich behavioral data sets from videos that were labeled and analyzed with DLC and SimBA to generate pose estimation behavioral classifiers. The process of obtaining these data sets was more efficient than manual annotation of 2 h videos across all behavioral sessions and revealed patterns of behavioral changes that were not detected with lever responses alone. The current methodology could be applied to reveal differences in quadrant time, orientation to levers or classifiers at different test times (short vs extended abstinence), and behaviors across different age groups (adolescent vs adult), sex (female vs male), or self-administration procedures (short, long, or dual social intake; Smith, 2012; Nicolas et al., 2019; Olekanma et al., 2023; Venniro et al., 2021; Popik et al., 2024).

## Synthesis

Reviewing Editor: Sam Golden, The University of Washington Decisions are customarily a result of the Reviewing Editor and the peer reviewers coming together and discussing their recommendations until a consensus is reached. When revisions are invited, a fact-based synthesis statement explaining their decision and outlining what is needed to prepare a revision will be listed below. The following reviewer(s) agreed to reveal their identity: David Dietz.

Dear Dr. Arguello,

Your manuscript has been read by two experts in the field. Both reviewers recognized the manuscripts clarity and detail in using video acquisition and DeepLabCut software to examine complex behaviors in an operant setting. Both reviewers also commended the well-written methodology and potential for future advancements.

However, Reviewer 1 suggests improvements such as enhanced figures, detailed screenshots, clarification on lighting conditions, and a deeper discussion on subject attrition and data interpretation to better understand self-administration behaviors. Reviewer 2, while appreciating the detailed methodology, raises concerns about the minimal "proof of principle" data collected from only three rats and the questionable utility of DeepLabCut when it failed to reveal new behaviors beyond those captured through conventional methods.

The collective feedback underscores the importance of expanding data presentation, refining analysis techniques, and clearly demonstrating the novel methodologies' utility to enhance the manuscript's impact on the field. Considerable additions have been noted as necessary for eventual manuscript approval by the reviewers. Based on these comments, please consider providing a revised manuscript for re-review. Below you will find the reviewers comments, please provide a point -by-point rebuttal addressing their concerns.

Reviewer 1:

The manuscript Sanbria et al., provides detailed instructions on developing, troubleshooting, and analysis steps for using video acquisition and then subsequently the software DeepLabCut to examine complex behaviors. The manuscript is very well written. Further, as this is methodology, the steps are easy to follow, even if these were not fully tested as part of this review. It is important to remark that such protocols are essential to fully understand "behaviors" such as self administration. For example, this is not one behavior but a suite of complex responses, a point to often forgotten in the analysis. There are some points to consider that would hopefully be helpful to the authors:

1) The figures are very basic and do not tell us much. Additional labeling would be very helpful (see below for some additional suggestions).

2) screen shots of successful video capture would be very helpful.

3) what degree of light is required or was used for capture here. Was this just the supplied house light? Presumably, the cue light which would only go on after a response was not the driving light source.

4) there is significant attrition in the subjects, which is not an issue. However, inclusion of what went wrong and inclusion of figures to demonstrate would be very helpful

5) The data of in quadrant presented during the last three days of SA is not surprising and in some ways on its own does not support the author's claims. What maybe more interesting is to plot days in which the discrimination active v. inactive were less discrete. This is true of animals that may not have met criteria.

6) Figures demonstrating what defines a quadrant, would be important.

7) why did the authors use FR1 throughout. In retrospect, the data for software analysis may have been more meaningful under a more strident schedule of reinforcement. Approach behaviors, goal vs. cue tracking, etc. Perhaps some thought about this could quickly be added to the discussion.

8) what is the rational of the explanation of the Context A and Context B? Is it just as simple as these were part of another ongoing study?

9) [added during reviewer discussion] I suggested that the authors show the behavior associated with acquisition rather than only the last three days. In this way, I was hoping they would have more data in terms of animals but also measures. This could include initial attentiveness to the cue vs. the lever, approach behaviors, ITI behaviors...etc. In this way we are able to assess if the the conclusions drawn from the three rats have more validity.

Reviewer 2:

This is a well-written manuscript and the authors' willingness to share their detailed approach to establishing DeepLabCut as a means to assess drug self-administration behavior is much appreciated. The methods were very detailed and appear to be sufficient for others to establish a similar approach. However, the "proof of principle" data that were included were minimal, and collected from just 3 rats. Further, the DeepLabCut approach, in this case, didn't add anything to the data that were already collected from the Med Associates chambers and therefore, the utility is questionable. Had DeepLabCut revealed behaviors that were not measurable from the chambers themselves, the potential impact of this manuscript would have been much greater.
